# Informational Support to Family Members of Intensive Care Unit Patients: The Perspectives of Families and Nurses

**DOI:** 10.5539/gjhs.v7n2p8

**Published:** 2014-09-25

**Authors:** Mina Gaeeni, Mansoureh A. Farahani, Naima Seyedfatemi, Nooredin Mohammadi

**Affiliations:** 1 School of Nursing and Midwifery, Iran University of Medical Sciences, Tehran, Iran; 2 Center for Nursing Care Research, School of Nursing and Midwifery, Iran University of Medical Sciences, Tehran, Iran

**Keywords:** Intensive Care Unit (ICU), family members, nurse, informational support, qualitative study

## Abstract

**Introduction:**

The receiving information about the patients hospitalized in the intensive care unit is classified among the most important needs of the family members of such patients. Meeting the informational needs of families is a major goal for intensive care workers. Delivering honest, intelligible and effective information raises specific challenges in the stressful setting of the intensive care unit (ICU). The aim of this qualitative study was to explain perspectives of families of Intensive Care Unit patients and nurses about informational support.

**Method:**

Using a conventional content analysis approach, semi-structured interviews were conducted with participants to explore their perspectives of providing informational support to families of ICU patients. A purposeful sampling method was used to recruit nineteen family members of thirteen patients hospitalized in the ICU and twelve nurses from three teaching hospitals. In general, 31 persons participated in this study. Data collection continued to achieve data saturation.

**Findings:**

A conventional content analysis of the data produced three categories and seven sub-categories. The three main categories were as followed, a) providing information, b) handling information and c) using information. Providing information had three sub-categories consisting of “receiving admission news”, “receiving truthful and complete information” and receiving general information. Handling information had two sub-categories consisting ‘keeping information” and “gradual revelation”. Lastly, using information has two sub-categories consisting of “support of patient” and “support of family members”.

**Conclusion:**

The results of this study revealed perspectives of families of Intensive Care Unit patients and nurses about informational support. It also determines the nurses’ need to know more about the influence of their supportive role on family’s ICU patients informing. In addition, the results of present study can be used as a basis for further studies and for offering guidelines about informational support to the families of the patients hospitalized in the ICU.

## 1. Introduction

As the first social institution, family bears culture, roles and special structure signifying physical, mental, social, spiritual and cultural health of its members. Any disorder in its parts will lead to the holistic disorder (Rabia Siahkali et al., 2010; Gavaghan, & Carroll, 2002; Li et al., 2003; Soltani, 2005). One of the changes affecting the family system is the time when one of its members is hospitalized. When the patient is hospitalized in the intensive care unit due to a serious illness or a life threatening event, the effect of this phenomenon becomes more severe (Maruiti, Galdeano, & Dias Farah, 2008). Patient’s critical situation and unclear prognosis can cause reactions such as fear, anxiety, physical and mental fatigue, hopelessness, disappointment and frustration in family members (Maruiti, Galdeano, & Dias Farah, 2008; Bephage, 2000; Tyrie, & Mosenthal, 2012; Yousefi, Karami, Moeini, & Ganji, 2012; Davidson, Jones, & Bienvenu, 2012). When one of the family members is hospitalized in the intensive care unit, some needs are aroused in the family (Obringer, Hilgenberg, & Booker, 2012). Receiving information, assurance, empathy and mental supports are among these needs (Sheaffer, 2010; Damboise, & Cardin, 2003; Bailey, Sabbagh, Loiselle, Boileau, & McVey, 2010). The need for information is universal and extremely important for all family members regardless of age, gender, socio-economic status and educational level (Mendonca & Warren, 1998; Bijttebier et al., 2000).

The results of a review prioritizing the needs of the family members of the patients in the intensive care unit show that receiving information about the patient is classified among the most important needs of such families (Naderi, Rajati, Yusefi, Tajmiri, & Mohebi, 2013). Also, some studies conducted in Australia, Belgium and China reported similar needs, such as feeling that the healthcare professionals care about the patient and being assured that their loved one is receiving the best possible care, receiving information once a day, obtaining honest answers to questions, being informed about the patient’s progress and expected outcome, and finally being informed about changes in the patient’s condition (Bijttebier, Vanoost, Delva, Ferdinande, & Frans, 2001; Burr, 1996). The results of a study also show that most of the stress and anxiety in patients’ family members is due to not acquiring enough information about prognosis, treatment and lack of familiarity with the environment and complicated equipments in the intensive care unit (Chien, Chiu, Lam, & Ip, 2006). Other studies have shown that the most important needs of relatives include obtaining information about the patient’s condition and about changes in his or her condition, and being assured that the best care is being given to the patient (Omari, 2009; Auerbach et al., 2005). Daly and Kloos (2008) also asserted that patients’ family members uncertainty and lack of information is an important factor in increasing their depression and anxiety. Informational support to the family members of the patients in the intensive unit equips them with better understanding the stressful situation and decreases their level of anxiety (Taylor, 2006). Using coping sources and strategies, giving information to the family members of the patients hospitalized in the ICU also helps them show better adaptation when confronted with such stressful conditions and can bring their expectations about their patients’ consequences closer to reality (Azoulay et al., 2002; Yaman, & Bulut, 2010; Lenz, & Perkins, 2000). In the time-constrained setting of the ICU, families need to receive information as quickly as possible, at admission of the patient and whenever the course of the disease changes abruptly (Azoulay et al., 2000).

Health care professionals that working in the intensive care unit are confronted with lots of ethical challenges because of the complications in giving care (Elpern, Covert, & Kleinpell, 2005). The family of the patients hospitalized in the intensive care unit often wants their questions to be answered honestly and comprehensibly. They also want to be informed about the changes in the clinical conditions of the patients as soon as possible (Davidson et al., 2007). In contrasts, because of the instability of patients clinical condition and family members mental imbalance, the health care professionals tends to give general and ambiguous information about the patient’s condition to protect the families against anxiety and worry (Miracle, 2006). Studies have shown that informational support from the family of patients will help them better adapt when they are confronted with stressful conditions. If the nurses give information to the family of the patient, they better adapt with stressful conditions and their expectations about the patient’s outcomes will be closer to reality.

In general, most studies performed about information needs of the families of patients in the ICU and were mostly in the form of quantitative research. There are only a few qualitative research on this topic, none of which focus on informational support from perspective these families and nurses. For the basis of family- orientated nursing practices, it is important to know more about families’ and nurses’ experiences of receiving and providing informational support during a critical illness. This knowledge will make it possible to better respond to families’ needs in the hospital setting. Therefore, this qualitative study has performed with aim of exploring perspectives of families of Intensive Care Unit patients and nurses about informational support.

## 2. Method

This qualitative study adopts a conventional qualitative content analysis approach. This method is one of the approaches of qualitative research and also of qualitative data analysis (Burns & Grove, 2005). Content analysis analyzes written, spoken or visual messages in which raw data are summarized and then categorized. In conventional content analysis categories and their names are taken from the data (Elo & Kyngäs, 2008).

### 2.1 Setting

The study was conducted in intensive care units of three teaching hospitals in Iran, where patients were admitted due to various medical conditions such as medical-surgical problems, neurosurgical problems and trauma. Each intensive care unit had eight beds and there was restriction for visitors to visit their patients.

### 2.2 Participants

Purposive sampling was used to identify nineteen family members of thirteen patients and twelve nurses who work in intensive care units. Family members were selected as following criteria: being the close family member’s (spouse, children, father, mother, sister and brother), being involved in patient’s follow and having rich experience of subject study, age older than 18 years, Iranian nationality and desire to participate in study. Selection criteria for nurses were at least one-year work experience in critical care unit, Iranian nationality and desire to participate in study.

### 2.3 Ethical Consideration

After approaching the research units and explaining the objectives of the study and also obtaining written consent of the participants, the researcher began to collect the data. Ethical principles such as autonomy of the participants, confidentiality and anonymity were considered throughout the study. Ethical approval was obtained from the Ethics Committee of Iran University of Medical Sciences, as well as from all the hospitals involved.

### 2.4 Data Collection

In this study, semi structured and face-to-face interviews were conducted with nineteen family members and twelve nurses. The duration of the interviews varied between 35 and 85 min with mean of 60 minutes. Interviews continued until data saturation was attained. In general, 31 interviews were done. Data collection process was continued to achieve data saturation and it took a fifteen- month period from Jun 2012 to Aug 2013. At the beginning of the interview, the families were asked a general question: What is your experience in achieve to information about your patient? Also, nurses were asked a general question: What is your experience in giving information to patient’s family? In order to attain more information, the interviews continued with probing questions. All interviews were recorded by voice recorder and then verbatim into transcripts word by word in order to analyze data.

### 2.5 Data Analysis

For data analysis, the researcher used conventional content analysis according to Graneheim and Lundman method (Graneheim & Lundman, 2004). This approach is usually appropriate when existing theory or research literature on a phenomenon is limited. Researchers used inductive category development, i.e., Avoided using preconceived categories, instead allowed the categories flow from the data. Researchers also immersed themselves in the data to allow new insights to emerge (Elo & Kyngäs, 2008).

According to content analysis process, at first each interview was read again and again carefully in order to gain a universal and primary understanding of the important under-lined statements. Then, meaning units about participants’ experiences of relationship existent in the interview text were determined. In the next phase, the meaning units were abstracted through condensation and were labeled as codes. Participants’ statements and implicit concepts were used for coding. Codes were compared for similarities and differences within the same interview and in different interviews, and then categorization of codes was done accordingly. In the next stage, categories and sub-categories were examined under supervision of expert supervisors who were experienced in qualitative analysis.

### 2.6 Rigor of the Study

Concepts of credibility, confirmability, auditability and transferability were used to measure the trustworthy of the data (Polit & Beck, 2013). Credibility of the data in this study was evaluated through member check, peer check and prolonged engagement. After the analysis, the participants were contacted and gave them a full transcript of their respective coded interviews with a summary of the emergent categories to approve interpretations of the researchers. An expert supervisor and two doctoral students of nursing checked the study process.

Prolonged engagement with the participants within the research field for a period of fifteen months helped us gain the participants’ trust and better understanding of their real world. We saved all evidences and documents securely to maintain audit ability. We carried out a thick description of the context adequately so that a judgment of transferability could be made by readers.

## 3. Results

From the nineteen family members participating in this study, there were 13 female and 6 male, The age of the family members varied between 19 and 49 years (mean of 34 years), and From the 12 nurses, there were 8 female and 4 male. The age average was 40.42± 2.16 years, and their work experience in critical care was ranged from 4 to 12 years. Three main categories emerged from the data, providing information, handling information and using information (Table 1).

**Table 1:** Categories and subcategories demonstrating the Informational Support to Family Members of Intensive Care Unit Patients

Categories	Subcategories
Providing. information	Receiving admission news
	Receiving truthful and complete information
	Receiving general information
Handling information	Keeping information
	Gradual revelation
Using information	Support of patient
	Support of family members

### 3.1 Providing Information

Families emphasized that access to information from healthcare professionals and families with common experiences made them hopeful. One of the categories clarified in this study was the providing information. This category has three subcategories: “receiving admission news”, “receiving truthful and complete information” and “receiving general information”.

#### 3.1.1 Receiving Admission News

Families began to obtain information from the time of receiving admission news about the patient in ICU. They mainly inquired whether their patient was alive, or not? They attempted to seek information from anywhere and anyone on the type and severity of the incident, and the reliability of the source was not important.

One of the patients’ mother said:

*“When I was told my son had an accident and taken to ICU, I thought my son was certainly dead and they were lying to me. I went to the hospital and asked hospital guards, service men, nurses and doctors what had happened to my son, I just wanted them to tell me the truth whether he was alive or not?”*[F8].

To gather information about the reason of patient’s admission in the ICU, nurses had different approaches about the family members.

A nurse having ten years of experience of working in the ICU said:

*“At the moment of patient’s hospitalization, I do my best to give the family a brief explanation, and make them prepared for the next step. For example, if the patient suffers from a severe illness, we’d better inform them gradually in order to make them prepared for bad news. I usually don’t try to make them disappointed or give them bad news in the first visit. Step by step, I tell them their patient is in coma, and he’s not unconscious, we have to wait for 24 or 48 hours to see what happens”* [N1].

Another nurse said:

*“Before giving any information, we ask what kind of relationship they have with the patient. It they have close relationship, depending on the situation, we gradually inform them. Our goal is to protect the family from any possible emotional damage in this stressful condition”* [N3].

#### 3.1.2 Receiving Truthful and Complete Information

Receiving truthful and complete information is the second subcategory of providing information category. After patients’ families pass the shock of bad news, they begin to seek more and valid information about their patients’ condition, so they seek reliable, specialist and honest people who they can have great and empathetic relationship with and that families can receive truthful and complete information from them. In fact, they were seeking safe, promising, valid and reliable sources in those unstable and critical conditions.

One of the patient’s wife said:

*“I believe they have to tell the truth. A person coming to ICU likes to hear the truth……….they have to tell the truth……..they rarely tell the truth. I don’t know why they don’t tell the truth”* [F18].

In this regard, many of the family members said that information must be offered complete and understandably. They also asserted that if the provided information meets their knowledge level, they can better understand it, and their interpretations will be sensible. This can decrease the level of their anxiety and concern.

In this regard, son of one of the patients said:

*“When I don’t have technical information, they have to explain it to me simply and understandably. Either they couldn’t explain, or I couldn’t understand what they said. I mean there was no common language between the physician, the nurse and patients’ family members”* [F16].

Misinterpretation of or misunderstanding the information besides not giving information understandably is what many family members complain about.

The same participant commented:

*“Sometimes, the interpretations were different. Unfortunately, because of the misinterpretation, sometimes we become more disappointed or more hopeful”* [F16].

What nurses mentioned emphasized that to provide honest and complete information for family members is conducted based on the related rules and regulation in this regard. Mental conditions, governing conditions in the ward, and the organization’s related rules and regulations are among the items nurses pointed out.

One of the nurses pointed out:

*“We can’t tell the truth, under some certain circumstances, to the patient’ family about his illness or its consequences is due to the situation in the ward. For example, if a patient suffers from brain death, and this news is suddenly exposed to his family, it’s probable for them to react involuntarily and make the ward and hospital agitated and mess which can badly affect other patients and their families. As a nurse, we can also inform the families just in nursing scope, and based on the hospital regulations, we aren’t allowed to give clear –cut information about medical scope to the families. In such cases, they are referred to see the doctors”* [N9].

#### 3.1.3 Receiving General Information

The last subcategory of providing information is receiving general information. As award with special conditions and advanced equipment, the ICU bears complicated features, and due to these features it has challenges special of its own. Therefore, the patients being hospitalized in this ward and their family members are involved in these challenges. Based on these challenges, the families are often required to receive information about the nature of the ICU, equipment attached to the patient, regulations about how to visit the patients, and so on. Most of the families participated in this study declared they tried to receive such information from different sources such as healthcare professionals, friends and relatives or from families with the same experience. Of course, they complained why the healthcare professionals talk less to them.

Concerning informational support one of the patient’s girl said:

*“When we were said our dad was going to be hospitalized in the ICU, we were upset. The word ICU makes you worried. In my opinion, ICU is the last step, but when we saw although in the ICU, he was getting better, we were glad. After 4 or 5 days when we came, we saw his mouth was shut and connected to equipment. We were afraid. I think when a patient is in the ICU, the overall information must be given to the families, and nurses must to some extent clarify the ICU’s regulation”* [F12].

Concerning being informed about the regulation of how to visit the patient, one of the patient’s wife believed:

*“It’d be better for nurses to understand our mental condition rather than insisting repeatedly that you are not allowed to visit the patient in the ICU. If a nurse treated us well and explained the condition to us, we’d better understand him/her better, too. Their good behavior could make us hopeful and confident”* [F15].

Regarding weaknesses in this aspect of informational support, the nurses participating in this study had their own specific reasons. Because of the patient’s critical condition and because saving the patient is a priority, most of the nurses preferred to support the family of the patient as a second priority. Nurses also pointed out they are not allowed to tell everything in the ward to the families of the patients, and giving information about some specific issues is in the charge of other healthcare professionals such as a doctors.

Another nurse said:

*“Although we concentrate to prioritize the patients and try to stabilize him/her, we understand the families; we know they’re upset and scared. I mean we react to them to help them not be excited and more stressed, because their excitement is disturbing for us. Therefore, we try to calm them down and give them in the first possible moment overall information about the ICU, equipment attached to the patient and patient’s condition”* [N3].

### 3.2 Handling Information

The second category of the study was handling information. This category has two subcategories: keeping information, gradual revelation. What attained from most of the interviews was that handling information in giving information can be one of the factors causing agitation and anxiety in such families.

#### 3.2.1 Keeping the Information

One of the subcategories of handling information is keeping the information. “control of information” by the health care providers was one of the strategies hidden in keeping information. There were many reasons to choose this strategy (control of information). Family’s mental and emotional condition, patient’s instability, physicians’ reluctancy in giving thorough information and constrictions related to the organization (special conditions of the intensive care unit) are a few to name.

In this regard, one of the nurses said:

*“Those colleagues telling the patient’s family members that the patient’s condition made no progress may be have responsive restrictions. For example, the physicians don’t like and allow the complete information to be accessible to the families”* [N9].

#### 3.2.2 Gradual Revelation

The second subcategory in handling information is gradual revelation. What came out of the participant’s statements in this report showed that family members wanted the Healthcare professionals to tell the true condition of their patients and inform them completely to alleviate their anxiety.

In this regard, one of the members of the patient’s family said:

*“When they answer indifferently and say madam: the condition is the same. Nothing changed. It makes me worried and stressed”* [F18].

The nurses participating in this study stated that it is not necessary, due to the mental and emotional condition of the family members and not anticipating prognosis and patient’s consequences, to give information completely and clearly.

In this regard, one of nurses said:

*“I believe if the family members of the patient’s are given the detailed situation of the patient, for example, your patient suffers from that illness or needs that diagnosis test, it may postpone the cure and make the family members protest that* *everything is not done perfectly. Therefore, we don’t make them involved in the treatment details”* [N1].

In this regard, nurses’ statements showed that incomplete information given by physicians and nurses to patients’ family members could cause confusion and ambiguity.

A nurse said:

*“May be the information that the nurse offers is not complete, and it may lead to contradictory information”*. [N11].

Nurses also announced that there was a contradiction between nurses and physicians in terms of giving information to patients\ them families. They believed that the main reasons of contradiction between nurses and physicians were lack of communication and coordination between physicians and nurses, physicians’ reluctance to provide information to patient’s family, and nurses’ de-motivation.

One of the nurses commented:

*“One of the reasons why the healthcare professionals aren’t functioning properly is because of personnel’s fatigue. The other reason is when the personnel see nobody appreciates what they’ve done and families are grateful to physicians, and the nurse role, as the most important factor in the patient care in the ICU, is not highlighted. The life of a patient in the ICU is at the hands of the nurses. This nurse’s unmotivatedness can affect the relation with the patients’ families”* [N3].

Nurses also announced that the lack of a uniform channel of giving information could be another factor in giving contradictory information.

One of the nurses stated:

*“If it’s feasible to have a system which person talks and the information is given through one channel to the patient’s family, maybe he can decide better for his patient “*[N5].

### 3.3 Using Information

The category of using information was another form of informational support. This category included two subcategory: “support of patient” and “support of family members”.

#### 3.3.1 Support of Patient

According to findings of this study, the families of patients in ICU made their decision about the current situation after they passed the critical period and obtained accurate information about their patients’ conditions and treatment outcome. Their decisions were based on how they could use information sources to provide better care and treatment for their patients.

The daughter of a patient with thyroid cancer said:

*“When I talked with my mother’s doctor, he thoroughly explained the situation and I became more hopeful, and when I went to hospital to visit my mother, I asked well-behaved nurses who took care of my mother more questions. I even learned how to perform some medical care from them (giving oxygen, performing suction, changing position, massaging, etc.). When I came out of the ICU, I felt empowered, I felt a power generated inside me that told me I could take care of my mom and that she would get well and back to normal life”* [F14].

On the one hand, the families of such patients needed information about the condition and outcome of the patient in order to overcome their worries and anxiety. On the other hand, in order to be prepared to care about their patients after dismissing from the ICU, they needed to be trained about care and how to use care equipment. Therefore, healthcare professionals, especially nurses, must help and guide the families by giving informational support to take better advantage from such information. Based on the findings of this study, most of the nurses emphasized on this aspect of their roles in protection of families. One of the experienced nurses said:

*“Since we provide calmness for the families and make them ensured, they pick us as their councilor. Wherever confronted with a problem with their patient, they come to us. It helps them not to be confused even after the patient is dismissed from the ICU, and if they’ve a question, they call us”*[N7].

#### 3.3.2 Support of Family Members

Another subcategories of using information was Support of family members. To attain a sustainable condition, better understanding and pave the way for a sound decision about issues related to the patient and other life things, the family members came to this conclusion to effectively react with the healthcare professional. Most families stated that support and the empathy from healthcare professionals could help them deal with the critical situation of their patients in ICU. Appropriate support from the healthcare professionals was a case repeated in the majority of families’ statements. They stated that the Kind and empathetic behavior of health providers created a positive attitude among family members and returned their life to normal state.

In this regard, one of the patients’ son commented:

*“The positive inner feelings made me able to comfort other family members of mine and to relax them”* [F5].

Also, most of the families stated:

“The personnel’s behavior is really effective in maintaining our morale; receiving right and proper answers, we leave the hospital with confidence and hope for our patient’s improvement.”

Concerning family support, one of the nurses also said:

*“In general, by understanding the patient’s condition, outcome and prognosis, the family members became calm and had a better behavior. I think it’s because of the appropriate relation with and support of the healthcare professionals to the families”* [N9].

## 4. Discussion

We conducted this qualitative study to explain informational support from perspectives of families of Intensive Care Unit patients and nurses. The results from data analysis showed three categories and seven subcategories. Three major categories that emerged from the data analysis in this study included: providing information, handling information and using information. In the case of providing information, the findings of current study demonstrated the access to information about the patients hospitalized in the intensive care unit was one of the most important needs of the family members of such patients. Providing information for families was a major goal for intensive care providers. These findings showed that informational support is a valuable issue from perspectives of families’ ICU patient and nurses. In this regard, literature review on needs of family members of intensive care unit (ICU) patients showed that information have crucial importance (Verhaeghe, Defloor, Van Zuuren, Duijnstee, & Grypdonck, 2005). In many studies, nurses also underestimate the importance that relatives attach to obtaining information (Kleinpell & Powers, 1992; Murphy, Forrester, Price, & Monaghan, 1992; Bijttebier et al., 2001). In aspect of receiving admission news families attempted to obtain information from the time of receiving admission news about the patient in ICU. They mainly inquired whether their patient was alive, or not? They tried to seek information from anywhere and anyone on the type and severity of the incident, and the reliability of the source was not important. In the study by Johansson and colleagues (2005), Most of the relatives experienced a life threatening situation during the period prior to arrival at the ICU. They described initial shock and chaos.

Another important aspect of providing information in this study was receiving truthful and complete information. Most of families participating in current study stated that, after they pass the shock of bad news, they begin to seek truth and valid information about their patients ‘condition. Delivering honest, intelligible and effective information raises specific challenges in the stressful setting of the intensive care unit (ICU). The challenge offered here is that patient’s family members expected the health care providers to behave with them honestly, and health care providers eschew giving thorough and honest information due to the reasons such as mental and emotional condition of the family members and patients’ unstable condition. In fact, they were dubious to choose to be or not to be honest. May be, if they told the truth, the family couldn’t tolerate, or because prognosis was not predictable, giving true and thorough information could create false hope and its consequent mental suffering in patient’s family. A study by Pergert and Lutzen (2012) showed that not telling the truth can be effective in order to protect people against mental effects such as decreasing hope and its consequent suffering. The most important need of the families is to receive real and appropriate information about their patients in order to be hopeful or disappointed, based on the current situation, about their patients. The importance of being provided with honest and reliable information about the patient’s state was demonstrated in some studies (Abbott, Sago, Breen, Abernethy, & Tulsky, 2001; Koller, 1991; Kleinpell, & Powers, 1992; Al-Hassan & Hweidi, 2004).

On the other hand, healthcare professionals believe, based on the patient’s prognosis and instability of the situation, overall and not to the point information should be given to the families. In this regard, the findings of two studies showed that, one of the major concerns of all family members is about recieving truthful and complete information that allows building realistic hope. However, the Healthcare professionals believed offering the minimum information strategy must be used in giving information to the families (Verhaeghe, van Zuuren, Defloor, Duijnstee, & Grypdonck, 2007; Serio, Kreutzer, & Witol, 1997).

In study by Fulbrook and colleagues (1999), most family members stated that they prefer to be told the truth even if it conflicts with their need for hope because ‘not knowing’ is the worst. Moreover, Bond and colleagues (2003) reported that families wanted their questions about the patient’s condition to be answered honestly and realistically.

Also, Studies done in this field showed that although families expected an information source to give them correct and complete information, physicians and nurses, because of patients unstable situation at the time of hospitalization in ICU and not having comprehensive information about his situation, could not give them complete information (Burr, 1998; Gavaghan, & Carroll, 2002).

The results of this study revealed, in addition, families need information about the reason of patient’s admission in the ICU and his/her condition, they required to receive general information about of the nature’s ICU, equipment attached to the patient and regulations about how to visit the patient. The families asserted this supplementary information helps them better understand of condition and reduces their anxiety. Also, they are hoping for better outcome about their love one. According to results of a study, the narratives showed the relatives severely affected by admission to ICU, because they were not prepared for this sudden event (Fridh, Forsberg, & Bergbom, 2009).

Also, Jones and Griffiths (1995) stated that Provision of confiding support, such as information, advice and guidance, by intensive care providers and social network, reduces symptoms of anxiety in families of ICU patients.

Handling information was another aspect of informational support to Family Members of Intensive Care Unit Patients. According to most of the interviews handling information in giving information could be one of the factors causing agitation and anxiety in such families. One of the strategies reported in handling information was keeping information which nurses wanted the most. Families tended to receive complete and understandable information, while the nurses believed giving general and ambiguous information would suffice. It was unnecessary, they believed, to give the information in detail. If the complete information in detail is given to families, it would be problematic for them. The nurses used control of information strategy. They were considerate what information to be given to which family members and how information to be offered. This process about giving information was sometimes in sharp contrast with the needs of the families. The results of a study showed that the health care providers confessed that families needed to be given information about changes in their patient’s condition and to be given with understandable information, but they cannot tolerate to receive all the information at once. Information, therefore, must be constantly and gradually offered (Bond, Draeger, Mandleco, & Donnelly, 2003).

It is inferred from the statements of the health care providers that by not telling the truth they did not mean to lie. Rather, they used gradual revelation offered in the handling information. In this situation again, a challenging condition occurred because of the different viewpoints that the health care providers and patients’ family members possessed. Each group had its own special reasoning. Therefore, it is difficult to indicate which method to be used in giving information to the families of the patients in the ICU, to tell or not to tell the truth. Based on the reasons extracted from participants’ (families and the health workers) attitude, telling the truth can be supportive in some circumstances and harmful in the others. Considering issues such as patient’s stability, families ’emotional situation and the existing conditions in the ICU and the hospital can be determining factors in the healthcare professionals’ ethical decisions in this regard.

In current study using information was another important aspect of informational support to ICU patients’ family. According to findings of this study, families using information sources to provide better care and treatment for their patients and they attempted to create a positive attitude among family members and return their life to normal state. In this dimension, Jussila’s study (2008) showed the power of family to provide required emotional support for the patient and other family member. Also, the results of a qualitative research showed that support of family members lead to feeling of calm and security (Johansson, Fridlund, & Hildingh, 2005).

Health professionals, especially nurses, should recognize dimensions of informational support to Family Members of Intensive Care Unit Patients. Such targeted support for families may be seen as an important intervention strategy to promote patient, as well as family, well-being. Moreover, there is a need for research on informational support to family members of Intensive Care Unit patients from perspective’s physicians. Also, in the current health care environment, nursing interventions to support family’s ICU patients, especially informational support, essential and research needs to develop and test such interventions.

### 4.1 Limitations

The study was conducted in three large referral hospitals in Iran; thus the findings should be interpreted in light of this context. Also, cultural beliefs of participants, especially families, may have influenced the interviews. However, sampling with maximum variation of different parts is one of the strengths of this study that increase its generalization to different fields.

## 5. Conclusion

This qualitative study revealed a small portion about informational support to the families of the patients in the ICU. The findings of this study can help nurses to think about informational needs more from the viewpoint of the family’s ICU patients. The results of this study showed three main categories and seven sub- categories in informational support to the families of the patients in the ICU (Table 1). The results revealed that the families wished that the staff would support them more by being present and available and by giving more information about the love one’s condition and treatment outcome. Participants also concurred about the importance of providing information about cause of ICU admission, nature’s ICU and existing equipments. On the other hands, most of disagreements were on how handling information.
